# SPRINT Treatment Among Adults With Chronic Kidney Disease From 2 Large Health Care Systems

**DOI:** 10.1001/jamanetworkopen.2024.53458

**Published:** 2025-01-07

**Authors:** Manjula Kurella Tamura, Mengjiao Huang, Jaejin An, Mengnan Zhou, Fang Niu, John J. Sim, Nicholas M. Pajewski, Sarah A. Gaussoin, June Li, Michelle C. Odden, Tara I. Chang, Vivek Charu, Maria E. Montez-Rath

**Affiliations:** 1 Division of Nephrology, Department of Medicine, Stanford University School of Medicine, Palo Alto, California; 2 Geriatric Research, Education and Clinical Center, VA Palo Alto, Palo Alto, California; 3 Department of Research and Evaluation, Kaiser Permanente Southern California, Pasadena; 4 Division of Nephrology and Hypertension, Kaiser Permanente Los Angeles Medical Center, Los Angeles, California; 5 Department of Biostatistics and Data Science, Wake Forest University School of Medicine, Winston-Salem, North Carolina; 6 Department of Epidemiology and Population Health, Stanford University School of Medicine, Palo Alto, California; 7 Quantitative Sciences Unit, Department of Medicine, Stanford University School of Medicine, Stanford, California; 8 Department of Pathology, Stanford University School of Medicine, Stanford, California

## Abstract

**Importance:**

It is unclear whether the effects of intensive vs standard blood pressure (BP) targets seen in clinical trials generalize to patients with chronic kidney disease (CKD) encountered in everyday practice due to differences in the distribution of cardiovascular risk factors and coexisting conditions.

**Objective:**

To evaluate whether the beneficial and adverse effects of intensive vs standard BP control observed in the Systolic Blood Pressure Intervention Trial (SPRINT) are transportable to a target population of adults with CKD in clinical practice.

**Design, Setting, and Participants:**

This comparative effectiveness study identified 2 populations with CKD who met the eligibility criteria for SPRINT between January 1 and December 31, 2019, in the Veterans Health Administration (VHA) and Kaiser Permanente of Southern California (KPSC). Baseline covariate, treatment, and outcome data from SPRINT were combined with covariate data from these populations to estimate the treatment effects in the target population, applying models that estimated outcomes using distributions in the trial. Analysis was performed between May 2023 and October 2024.

**Main Outcomes and Measures:**

The main outcomes were major cardiovascular events, all-cause death, cognitive impairment, CKD progression, and adverse events at 4 years.

**Results:**

A total of 85 938 patients (mean [SD] age, 75.7 [10.0] years; 81 628 [95.0%] male) from the VHA and 13 983 patients (mean [SD] age, 77.4 [9.6] years; 5371 [38.4%] male) from KPSC were included. Compared with 9361 SPRINT participants (mean [SD] age, 67.9 [9.4] years; 6029 [64.4%] male), these patients were older, had less prevalent cardiovascular disease, higher albuminuria, and used more statins. The associations of intensive vs standard BP control with major cardiovascular events, all-cause death, and adverse events were transportable from the trial to the VHA and KPSC populations; however, the trial’s effects on cognitive and CKD outcomes were not transportable in 1 or both clinical populations. Intensive vs standard BP treatment was associated with lower absolute risks for major cardiovascular events at 4 years by 5.1% (95% CI, −9.8% to 3.2%) in the VHA population and 3.0% (95% CI, −6.3% to 0.3%) in the KPSC population and higher risks for adverse events by 1.3% (95% CI, −5.5% to 7.7%) in the VHA population and 3.1% (95% CI, −1.5% to 8.3%) in the KPSC population.

**Conclusions and Relevance:**

In this comparative effectiveness study, the reduction in fatal and nonfatal cardiovascular end points and the increase in adverse events observed in SPRINT were largely transportable to trial-eligible CKD populations from clinical practice, suggesting benefits of implementing intensive BP targets.

## Key Points

**Question:**

Do the beneficial and adverse effects of intensive vs standard blood pressure (BP) control observed in the Systolic Blood Pressure Intervention Trial (SPRINT) generalize to adults with chronic kidney disease (CKD) from clinical practice?

**Findings:**

In this comparative effectiveness study of 99 921 SPRINT-eligible adults with CKD from 2 health care systems using transportability analysis to estimate mean treatment effects, intensive vs standard BP control was associated with comparable relative benefits and larger absolute benefits than those observed in SPRINT.

**Meaning:**

These results suggest potential population-level benefits of intensive BP targets in SPRINT-eligible adults with CKD.

## Teaser

This comparative effectiveness study evaluates whether the beneficial and adverse effects of intensive vs standard blood pressure control observed in the Systolic Blood Pressure Intervention Trial (SPRINT) are transportable to a target population of adults with chronic kidney disease from clinical practice.

## Introduction

Reducing morbidity attributable to hypertension is a pillar of chronic kidney disease (CKD) management. Guidelines from the Kidney Disease Improving Global Outcome (KDIGO) recommend treatment of hypertension to a target systolic blood pressure (BP) of less than 120 mm Hg when tolerated.^1^ This recommendation is based on the Systolic Blood Pressure Intervention Trial (SPRINT) and its prespecified subgroup analyses in participants with CKD.^2,3^ SPRINT found that treatment to a systolic BP less than 120 mm Hg vs less than 140 mm Hg reduced mortality, cardiovascular events, and mild cognitive impairment; increased certain adverse events, such as acute kidney injury; and had no effect on CKD progression.^2,3,4^

One of the main points of controversy concerning the KDIGO BP target is the uncertain generalizability of SPRINT to adults with CKD in clinical practice.^5,6^ Studies generalizing SPRINT treatment to the US population using NHANES data are limited by the small number of individuals with advanced CKD in the NHANES sample.^7^ Furthermore, although randomized trials have strong internal validity, they may not reflect the risks and benefits of treatment in clinical populations. Trial eligibility criteria and enrollment procedures result in study populations with different clinical characteristics than patients in routine practice.^8,9^ When the effect of treatment depends on baseline covariates (effect modification), and the distribution of such covariates differs between the trial sample and the target population, the treatment effect estimated in the trial sample will be biased or will not generalize to the target population. Traditional, 1-variable-at-a-time subgroup analyses in trials often have limited power to detect effect modification and usually explore effect modification on the relative but not the absolute scale. Additionally, such approaches do not provide patient-centered estimates of treatment effects because patients have many attributes that simultaneously affect the outcome and benefits of treatment.^10^

Prespecified subgroup analyses in SPRINT by CKD status did not show evidence of effect modification on the main cardiovascular end points.^3^ However, a post hoc analysis of SPRINT,^11^ a large meta-analysis,^12^ and observational studies^13^ suggest the cardiovascular benefits of intensive BP treatment may be attenuated in the population with advanced CKD. These observations suggest smaller potential benefits of intensive BP treatment and, coupled with the absence of information about adverse events in meta-analyses, raise concerns about the net benefit of intensive BP targets in adults with CKD. Presently, the field relies mainly on expert commentaries to contextualize evidence rather than objective assessments of generalizability, contributing to uneven implementation.^5,6^

These challenges may be informed empirically by transporting trial results to target populations. Transportability analyses apply treatment effect estimates generated from the trial sample to a target population of interest, accounting for potential differences in the distribution of baseline covariates. As such, these analyses can estimate the effects of treatment in a clinical population while preserving randomization, thereby informing how evidence is applied in broader populations.^14,15,16^ In this study, we sought to estimate the outcomes associated with treating hypertension to a systolic BP less than 120 mm Hg vs less than 140 mm Hg in 2 SPRINT-eligible clinical populations with CKD from the Veterans Health Administration (VHA) and Kaiser Permanente of Southern California (KPSC).

## Methods

This comparative effectiveness study was approved by the institutional review boards at Stanford University, the VA Palo Alto Health Care System’s Office of Research and Development, and KPSC. The study received a waiver of informed consent because it was considered minimal risk. This study follows the International Society for Pharmacoeconomics and Outcomes Research (ISPOR) reporting guideline for comparative effectiveness research.

### Data Sources

This study used 3 independent data sources: publicly available data from SPRINT and 2 distinct electronic health record databases used to define various target populations of interest. We followed the recommended steps by Ling et al^17^ to transport the clinical trial result into the target populations of interest (eFigure 1 in Supplement 1).

### SPRINT Population

The design and primary outcomes in SPRINT have been previously reported.^2^ SPRINT was an open-label nonblinded randomized clinical trial that enrolled adults without diabetes and with hypertension and elevated cardiovascular risk, indicated by the presence of CKD or other cardiovascular risk factors. Participants were randomly assigned to a treatment target of systolic BP less than 120 mm Hg vs less than 140 mm Hg. There were 9361 participants in the trial and 2646 participants in the CKD subgroup.^3^ In SPRINT, participants were followed from randomization until dropout, death, or trial termination (August 20, 2015), with a median follow-up of 3.3 years.^18^ For the cognitive outcomes, the end point was ascertained after unblinding of the intervention. The final date for follow-up of cognitive outcomes was July 22, 2018.^4,18^

We used the entire trial population except for the CKD progression outcome, which was modeled only in participants with an estimated glomerular filtration rate (eGFR) less than 60 mL/min/1.73 m^2^ based on the Modification of Diet in Renal Disease equation. We used this approach because the main inferences from the trial are based on the analyses in the total trial population, and the CKD subgroup analyses were underpowered. To model the CKD progression outcome, we restricted the sample to the SPRINT CKD subgroup.

### CKD Target Populations

As described in our previous work,^19^ we identified 2 clinical populations with CKD and hypertension from the VHA and KPSC between January 1 and December 31, 2019. From these cohorts, we applied the SPRINT inclusion and exclusion criteria (eFigure 2 and eFigure 3 in Supplement 1) to identify SPRINT-eligible adults constituting the 2 main target populations. Among these, 2% of individuals in the VHA CKD and hypertension population and 5% of individuals in the KPSC CKD and hypertension population were missing information required to determine eligibility into the SPRINT trial. In our previous work,^19^ we used multiple imputation to determine eligibility for those individuals. Due to the high level of correlation between the imputed datasets, we used 1 imputed dataset to define the target population.

### SPRINT Trial Outcomes

The outcomes used in the analysis are those defined in SPRINT: the primary outcome was a composite of myocardial infarction, acute coronary syndrome, stroke, heart failure, or death from cardiovascular causes, based on adjudication). The secondary outcomes included all-cause mortality, individual major adverse cardiovascular events, adjudicated dementia and mild cognitive impairment, CKD progression (defined as the composite of a 50% decrease in eGFR or development of kidney failure requiring chronic dialysis or kidney transplantation), and individual components of the CKD outcome. We also ascertained participant-reported serious adverse events, including hypotension, syncope, bradycardia, electrolytes abnormality, injurious fall, and acute kidney injury.^3,4,18^ We did not ascertain outcomes in the target populations and only used SPRINT trial participants when modeling the outcomes.

### Effect Modifiers

From previously published literature,^10,20^ we identified variables captured in both the trial and target populations considered to be potential effect modifiers of the treatment on outcomes and those associated with outcomes. These included baseline age (continuous), sex (female vs male), self-identified race and ethnicity (Hispanic, non-Hispanic Black, non-Hispanic White, and other [eg, American Indian or Alaska Native, Asian, and Hawaiian or Pacific Islander]), cardiovascular disease history (presence of 1 of the 7 *International Classification of Diseases, Ninth Revision *(*ICD-9*) or* International Statistical Classification of Diseases and Related Health Problems, Tenth Revision *(*ICD-10*) and/or Current Procedural Terminology –defined cardiovascular conditions, including myocardial infarction, acute coronary syndrome, coronary artery bypass surgery, percutaneous coronary intervention, carotid endarterectomy, peripheral vascular disease with revascularization and abdominal aortic aneurysm repair [eTable 1 in Supplement 1]) in the past 24 months (yes vs no), systolic BP, diastolic BP, urine albumin to creatinine ratio (UACR), eGFR, Framingham Risk Score, body mass index (BMI; calculated as weight in kilograms divided by height in meters squared), number of BP medications, high-density lipoprotein cholesterol (HDL), low-density lipoprotein cholesterol (LDL), ever smoker (yes vs no), and statin use (yes vs no). Race and ethnicity were primarily recorded as descriptive variables.

### Statistical Analysis

We compared baseline characteristics between the SPRINT population and the VHA and KPSC target populations using standardized mean differences. Standardized mean differences greater than 0.1 indicate meaningful differences between the trial sample and the target population.^17,21^

To obtain a comparable estimand for the analysis, we reestimated the mean treatment effect in SPRINT by calculating the counterfactual risk ratio (RR) at 4 years for each outcome.^22^ For this purpose, we estimated an individual’s probability of each outcome after fitting a flexible parametric survival model with the treatment group as the only variable in the model.^23^ Using a standardization approach, we computed the estimated risk in the intensive group and in the standard group by assuming all SPRINT individuals were in the intensive treatment group and in the standard treatment group, respectively. The RR is the ratio of the estimated risks. We computed 95% bootstrap CIs using 1000 samples run separately for each outcome.

We used the outcome-based approach to transport results from a randomized trial to the VHA and KPSC CKD population who met the trial’s eligibility criteria.^17,24,25^ The general analytical approach uses a counterfactual framework and is described in more detail in eMethods 1 and eFigure 1 in Supplement 1).^24^ Under this framework, we fit a model, conditional on baseline covariates, using participants in SPRINT randomized to intensive treatment. The fitted model is then applied to the target population to estimate the risk of treating the population to a target systolic BP less than 120 mm Hg. Similarly, we fit a model on participants randomized to standard treatment to estimate the risks of treating the population to a target systolic BP less than 140 mm Hg. Comparisons (differences or ratios) of the counterfactual effects estimate the treatment effect in the target population. We applied this approach to each outcome separately. We prespecified that trial results transport to the target population if the treatment estimate in the target population fell into the 95% CI for the treatment effect estimated using the trial sample (estimate agreement).^17^

Finally, we estimated the risk difference and corresponding 95% CIs by modeling the incidence rate for each outcome under intensive treatment minus the incidence rate under standard treatment. The number needed to treat (NNT) or the number needed to harm (NNH) is the additive inverse of the risk difference. To address whether SPRINT transports to the subgroup with more advanced CKD, we transported the trial to the target population with an eGFR less than 45 mL/min/1.73 m^2^, and separately, those with an eGFR less than 30 mL/min/1.73 m^2^.

All analyses were performed in R software version 4.1.2 (R Project for Statistical Computing). Multiple imputation was performed using the package mice version 3.16.0.^26^ The random forest model was estimated using the package randomForestSRC version 3.2.2.^27^ Analysis were performed between May 2023 and October 2024.

During the analysis, missing covariates were imputed using the fully conditional specification method with 10 imputation sets consisting of trial and target data. Each missing variable was imputed conditioning on all other covariates in addition to indicators for selection, treatment, and the Nelson-Aalen estimator of the primary outcome.^17,28^ We estimated the final transported RR as the mean of the estimated RRs using the 10 imputed datasets. We calculated 95% bootstrap CIs based on 500 samples.^24,29^ We applied the analytical approach within each bootstrap sample.

## Results

### Population Characteristics

We identified 85 938 adults (mean [SD] age, 75.7 [10.0] years; 81 628 [95.0%] male) with CKD in the VHA and 13 983 adults (mean [SD] age, 77.4 [9.6] years; 5371 [38.4%] male) with CKD in KPSC who met the SPRINT eligibility criteria (Table). Compared with 9361 SPRINT participants (mean [SD] age, 67.9 [9.4] years; 6029 [64.4%] male), adults in the VHA CKD and hypertension cohort were older, more likely to be male, and more likely to be White or other race or ethnicity. They were less likely to have cardiovascular disease and more likely to smoke and use statins compared with the SPRINT population. They also had a higher Framingham risk score, higher UACR, lower HDL, and lower LDL and were prescribed fewer BP medications (Table). Compared with the SPRINT population, adults in the KPSC CKD and hypertension cohort were older, more likely to be female, and more likely to be Hispanic or other race or ethnicity. They were less likely to have cardiovascular disease or smoke and more likely to use statins compared with the SPRINT population. They also had a higher UACR, lower diastolic BP, and lower LDL and were prescribed fewer BP medications (Table).

**Table.** Baseline Characteristics of SPRINT Participants and SPRINT-Eligible Populations With Chronic Kidney Disease From the Veterans Health Administration and Kaiser Permanente of Southern California

Characteristic	Participants, No. (%)			Standardized difference	
	SPRINT (N = 9361)	SPRINT-eligible adults with CKD			
		VHA (N = 85 938)	KPSC (N = 13 983)	Trial vs VHA	Trial vs KPSC
Age, mean (SD), y	67.9 (9.4)	75.7 (10.0)	77.4 (9.6)	−0.80	−0.99
Sex					
Female	3332 (35.6)	4310 (5.0)	8612 (61.6)	0.82	−0.54
Male	6029 (64.4)	81628 (95.0)	5371 (38.4)		
Race and ethnicity^a^					
Hispanic	984 (10.5)	3142 (3.7)	2273 (16.3)	0.27	−0.17
Non-Hispanic Black	2820 (30.1)	17690 (20.8)	2713 (19.4)	0.21	0.25
Non-Hispanic White	5399 (57.7)	58911 (69.4)	7830 (56.0)	−0.25	0.03
Other^b^	158 (1.7)	5151 (6.1)	1167 (8.3)	−0.23	−0.31
Cardiovascular disease	1165 (12.4)	3194 (3.7)	293 (2.1)	0.32	0.41
Systolic BP, mm Hg^a^	139.7 (15.6)	141.0 (10.2)	138.7 (7.9)	−0.15	0.08
Diastolic BP, mm Hg^a^	78.1 (11.9)	76.7 (9.7)	71.2 (11.3)	0.13	0.60
UACR, mg/g^a^	9.5 (5.6 to 21.4)	18.0 (7.0 to 62.4)	20.1 (7.1 to 87.1)	−0.10	−0.15
eGFR, mL/min/1.73 m^2^^c^					
Mean (SD)	73.3 (19.4)	42.6 (166.2)	48.4 (8.8)	1.64	1.66
≥60.0	7030 (75.1)	NA	NA	NA	NA
45.0-59.9	1483 (15.8)	61460 (71.5)	9751 (69.7)	−1.36	−1.30
30.0-44.9	688 (7.3)	20920 (24.3)	3633 (26.0)	−0.48	−0.52
20.0-29.9	139 (1.5)	3558 (4.1)	599 (4.3)	−0.16	−0.17
<20.0	21 (0.2)	NA	NA	NA	NA
Framingham Risk Score^a^	20.1 (10.8)	43.4 (17.9)	19.8 (11.0)	−1.57	0.02
BMI^a^	29.8 (5.6)	28.5 (5.4)	28.4 (6.0)	0.24	0.24
BP medications, mean (SD), No.	1.8 (1.0)	1.4 (1.2)	1.6 (1.2)	0.39	0.15
HDL, mg/dL^a^	52.9 (14.5)	47.2 (14.1)	54.3 (15.5)	0.39	−0.10
LDL, mg/dL^a^	112.4 (35.1)	96.6 (34.2)	93.9 (33.3)	0.46	0.53
Ever smoked^a^	5186 (55.5)	60394 (70.3)	6033 (43.1)	−0.31	0.25
Statin use	2750 (29.4)	32384 (37.7)	7803 (55.8)	−0.18	−0.55

Abbreviations: BMI, body mass index (calculated as weight in kilograms divided by height in meters squared); BP, blood pressure; CKD, chronic kidney disease; eGFR, estimated glomerular filtration rate; HDL, high-density lipoprotein; KPSC, Kaiser Permanente of Southern California; LDL, low-density lipoprotein; NA, not applicable; SPRINT, Systolic Pressure Intervention Trial; UACR, urine albumin to creatinine ratio; VHA, Veterans Health Administration.

SI conversion factor: To convert HDL and LDL to millimoles per liter, multiply by 0.0259.

^a^ Missing values observed in the variables were mostly less than 3%, except for 5.7% in LDL in VHA, and 18.8% in UACR in KPSC. Missing values were imputed using multiple imputation when we conducted the transportability analysis.

^b^ Includes American Indian or Alaska Native, Asian, and Hawaiian or Pacific Islander.

^c^ eGFR was calculated without a race adjustment.

### Cardiovascular, Mortality, and Cognitive Outcomes

**Figure 1.**
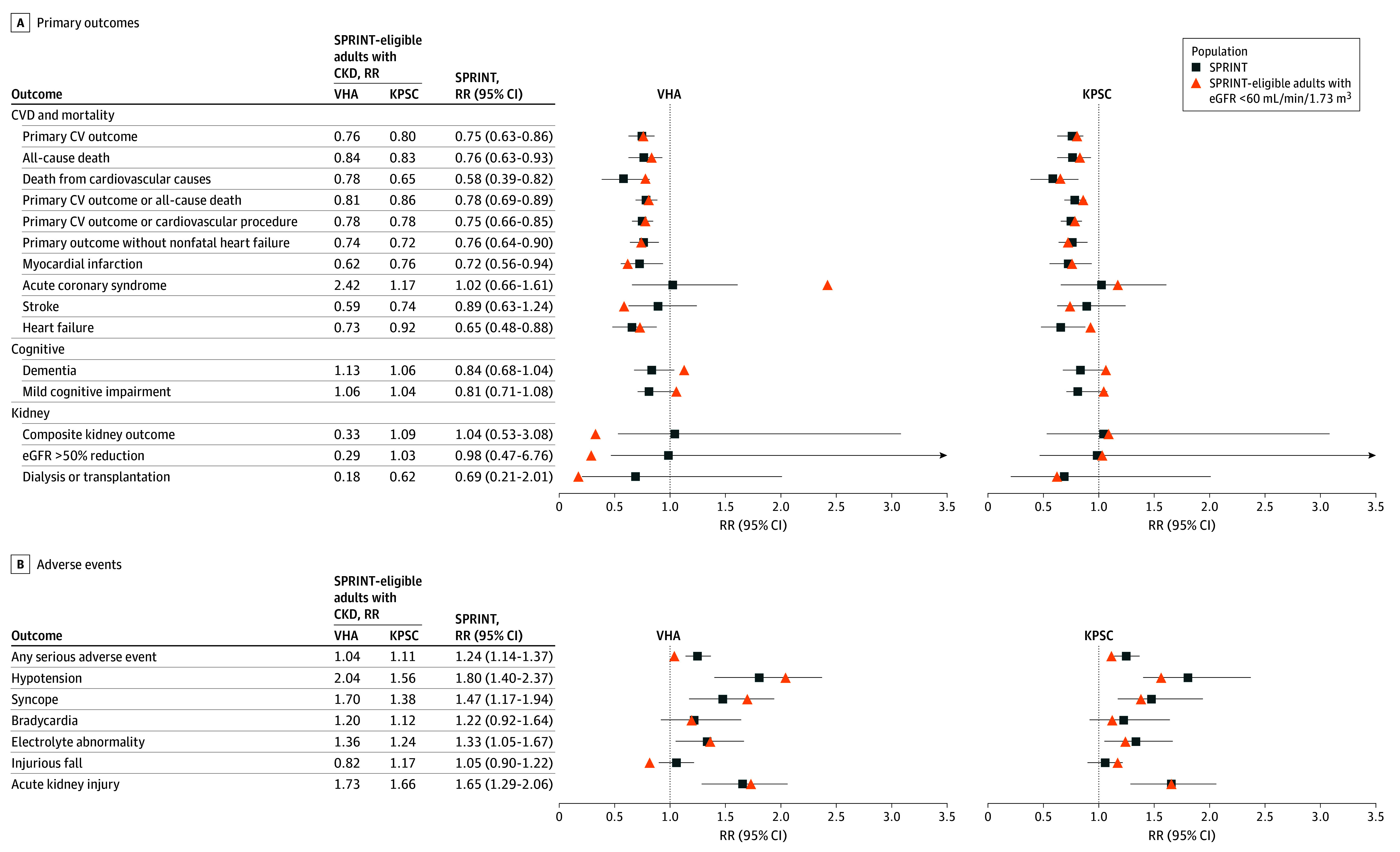
Risk Ratios (RRs) for the Primary and Secondary Outcomes and Adverse Events at 4 Years With Intensive vs Standard Blood Pressure Treatment in Adults With Chronic Kidney Disease, Stratified by Health System Transportability is based on estimate agreement, ie, the target mean treatment risk ratio falls within the 95% CI of the trial mean treatment risk ratio. CV indicates cardiovascular; CVD, CV disease; eGFR, estimated glomerular filtration rate; KPSC, Kaiser Permanente of Southern California; SPRINT, Systolic Pressure Intervention Trial; VHA, Veterans Health Administration.

First, using SPRINT data, we replicated the sample mean treatment effect in the trial population for the primary and secondary cardiovascular, cognitive, and kidney outcomes and adverse events (eTable 2 in Supplement 1). Next, we assessed treatment associations in the primary target populations, SPRINT-eligible adults with CKD in the VHA and KPSC. We found that treatment associations for cardiovascular events and mortality were transportable (ie, the treatment effect estimate in the target population fell within the 95% CI for the treatment effect estimated using the trial sample), with a few exceptions (Figure 1A). We estimated a larger benefit associated with intensive BP treatment for the secondary end points of stroke in the VHA population compared with the trial estimate. Conversely, we estimated a significant risk for harm associated with intensive BP treatment for the secondary end point of acute coronary syndrome events in the VHA population, whereas the trial estimated no significant difference. In the KPSC population, we estimated no benefit associated with intensive BP treatment for the secondary end point of heart failure, whereas the trial estimated a significant benefit. In both the VHA and KPSC populations, treatment effects on the primary cognitive outcome of dementia were not transportable. Treatment effects on mild cognitive impairment were transportable in the target populations.

### CKD Progression and Adverse Events

We found that the effects of intensive BP treatment on CKD progression were transportable to the KPSC population but not to the VHA population, ie, the treatment effect estimated in the KPSC population, but not in the VHA population, fell within the 95% CI for the treatment effect estimated using the trial sample. In the VHA population, we found evidence of a larger benefit associated with intensive BP treatment for the composite kidney end point and the 50% reduction in eGFR end point; however, the CIs for these estimates were wide and included the possibility of no benefit (eTable 2 in Supplement 1). We found that treatment associations with adverse events were transportable, except for an estimated lower risk for any fall-related adverse events in the VHA population compared with the trial estimate (Figure 1B).

### Supplementary Analyses

**Figure 2.**
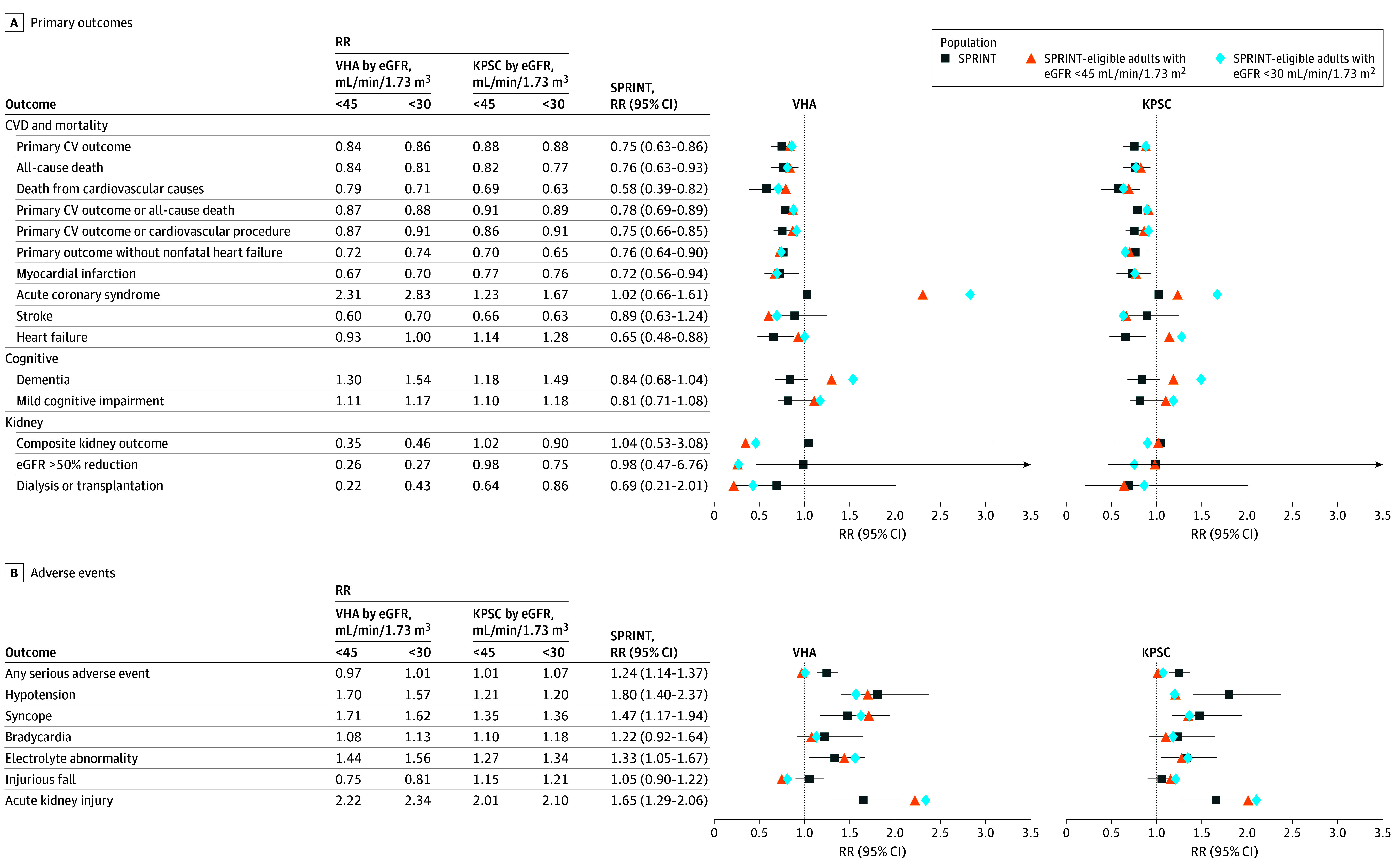
Risk Ratios (RRs) for the Primary and Secondary Outcomes and Adverse Events at 4 Years With Intensive vs Standard Blood Pressure Treatment in Adults With Advanced Chronic Kidney Disease, Stratified by Health System Kaiser Permanente of Southern California (KPSC) and Veterans Health Administration (VHA) populations are further divided by estimated glomerular filtration rate (eGFR). Transportability is based on estimate agreement, ie, the target average treatment risk ratio falls within the 95% CI of the trial mean treatment risk ratio. CV indicate cardiovascular; CVD, CV disease; SPRINT, Systolic Pressure Intervention Trial.

Treatment effects were transportable to the target population subgroup with advanced CKD, with a few important exceptions. Among the subgroup with an eGFR less than 30 mL/min/1.73 m^2^, we estimated a higher risk for the secondary end points of acute coronary syndrome, heart failure (KPSC only), and dementia and mild cognitive impairment with intensive vs standard BP treatment (Figure 2A; eTable 3 in Supplement 1), whereas the trial estimated a lower risk or no difference in these outcomes. We also estimated a higher risk for acute kidney injury and attenuated risks for hypotension (KPSC only) and injurious falls (VHA only) with intensive BP treatment (Figure 2B; eTable 3 in Supplement 1) compared with the effect estimated in the trial population. A similar pattern was observed when we assessed associations in the larger subgroup of adults with eGFR less than 45 mL/min/1.73 m^2^ (eTable 3 in Supplement 1).

### Absolute Risk Reductions

**Figure 3.**
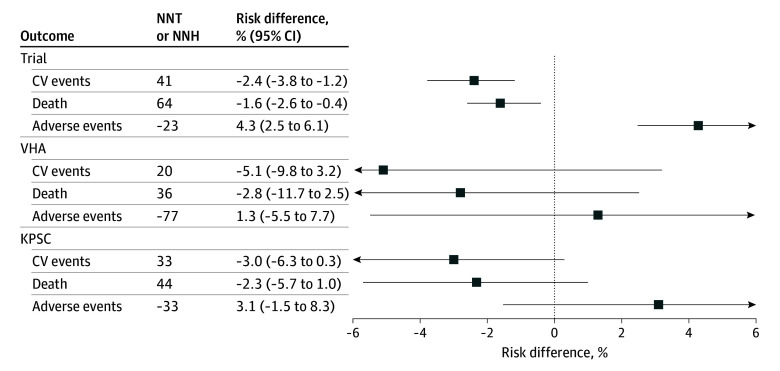
Risk Difference for Intensive vs Standard Blood Pressure Treatment in the Primary Cardiovascular Outcome, All-Cause Death, and Serious Adverse Events in the Trial, Veterans Health Administration (VHA) Chronic Kidney Disease (CKD), and Kaiser Permanente of Southern California (KPSC) CKD Populations CV indicates cardiovascular; NNH, number needed to harm; NNT, number needed to treat.

On the absolute scale, there were similar or larger risk reductions in major cardiovascular events and mortality in the VHA CKD and KPSC CKD populations compared with the trial estimates, while the estimated risk increase in adverse events in the VHA and KPSC populations was similar or smaller than the trial estimates (Figure 3; eTable 4 in Supplement 1). Intensive vs standard BP treatment was associated with lower absolute risks for major cardiovascular events at 4 years by 5.1% (95% CI, −9.8% to 3.2%) in the VHA population and 3.0% (95% CI, −6.3% to 0.3%) in the KPSC population and higher risks for adverse events by 1.3% (95% CI, −5.5% to 7.7%) in the VHA population and 3.1% (95% CI, −1.5% to 8.3%) in the KPSC population. In the VHA CKD population, we estimated the NNT with intensive vs standard BP control over 4 years to prevent 1 cardiovascular event or cardiovascular death was 20, the NNT to prevent all-cause death was 36, and the NNH to cause 1 serious adverse event was 77. In the KPSC CKD population, we estimated the NNT with intensive vs standard BP control over 4 years to prevent 1 cardiovascular event or cardiovascular death was 33, the NNT to prevent 1 all-cause death was 44, and the NNH to cause 1 serious adverse event was 33.

## Discussion

This comparative effectiveness study estimated the population mean association of intensive vs standard BP treatment with outcomes in 2 SPRINT-eligible clinical populations with CKD. We found that the reduction in cardiovascular and mortality end points and increase in adverse events observed in SPRINT were largely transportable to the clinical CKD populations. However, there were a few important exceptions. The associations of intensive BP treatment with cognitive and CKD progression outcomes were not transportable to clinical CKD populations. Additionally, while the associations of intensive BP treatment with the primary cardiovascular and mortality end point and composite adverse event end point were transportable to clinical populations of adults with an eGFR less than 30 mL/min/1.73 m^2^, there were also higher risks associated with intensive BP treatment for several outcomes and adverse events in this subgroup, including acute coronary syndrome, heart failure, dementia and mild cognitive impairment, and acute kidney injury. This analysis demonstrates the potential population-level benefits of intensive BP targets in SPRINT-eligible adults with CKD and highlights risk-benefit trade-offs in adults with advanced CKD.

Meta-analyses have attempted to overcome the limited power of CKD subgroup analyses in trials by pooling data for participants with CKD across multiple BP trials. While these approaches provide important evidence, they often omit analyses of adverse events due to nonuniform definitions and do not directly address the effects of treatment in nonenrolled populations. The lack of information about adverse events may be particularly important, as studies suggest the primary driver of patients’ preference for treatment is the type and severity of adverse events rather than the magnitude of benefit.^30^ In a meta-analysis of CKD participants from 18 BP target trials, including SPRINT, an intensive vs standard BP target was associated with lowering the risk for mortality by 14%.^31^ A separate meta-analysis of 123 BP-lowering trials found that lowering BP by 10 mm Hg was associated with reducing the risk for mortality in participants with CKD by 19%.^12^ In the same analysis, the associations of BP lowering with major cardiovascular and heart failure events were attenuated among participants with CKD vs those without CKD.^12^

Our results complement these meta-analyses and the SPRINT CKD subgroup analysis in 3 important aspects. First, we found that the trial and clinical populations differed in several characteristics, including age, sex, prior cardiovascular disease, smoking history, and statin use, and the clinical populations were also markedly different from each other. Nevertheless, the mean treatment effects on the primary outcomes in both clinical populations were consistent with those observed in the trial. This observation suggests that most baseline covariates in the models are not effect modifiers for the outcomes on the relative scale, supporting the rationale for BP targets based on absolute risk rather than clinical characteristics.^12,32^ Second, our analysis evaluates multiple secondary outcomes and adverse events. By including a broader set of outcomes, our analysis assesses benefits relative to the risk of harm. Third, this analysis provides insights into the generalizability of BP targets to patients with advanced CKD, a subgroup with limited representation in SPRINT. By demonstrating that intensive BP control had similar associations with the primary cardiovascular and mortality end points but higher risks for certain adverse events in this subgroup, these findings may help to reconcile conflicting or inconclusive results from previous trials and observational studies of BP control.

For similar estimated relative risk reductions between the trial and clinical CKD target populations, there were larger projected absolute benefits in the CKD target populations and similar or smaller absolute harms. This translated into a more favorable benefit-to-harm ratio in the VHA and KPSC CKD populations compared with the trial population. Using the VHA CKD population as an example, for every 4 patients who have a cardiovascular event prevented with intensive BP treatment, 1 patient would experience a serious adverse event. In the KPSC CKD population, for every 1 patient who has a cardiovascular event prevented with intensive BP treatment, 1 would experience a serious adverse event. In contrast, in the trial population, for every 3 patients who have a cardiovascular event prevented, 5 would experience a serious adverse event.

### Limitations

This study has some limitations, and several aspects of the analyses should be considered when interpreting these results. First, transportability analyses assume that patient characteristics associated with enrollment and heterogeneous treatment effects are measured. While we accounted for multiple baseline characteristics, our study could not account for characteristics that are not well-documented in electronic health records, such as limited life expectancy. If these unmeasured factors act as modifiers of the treatment or risk of outcome, then our estimates could be biased. Similarly, extending the analyses to groups excluded from SPRINT, such as adults with diabetes, assumes that these characteristics are not modifiers. Second, we assessed transportability based on estimate agreement, which is a less stringent requirement than regulatory agreement, replication of the direction, and statistical significance of the trial effects.^33^ However, the overall conclusions of this analysis would not change had we used regulatory agreement as the criterion for transportability. Third, the CIs for transportability estimates depend on the frequency of outcomes observed in the trial. Transportability analyses cannot overcome limited power for infrequent outcomes, such as the CKD progression outcome in SPRINT. Along these lines, some have cautioned against looking for effect modification when the treatment effect for an outcome, such as CKD progression, is null.^34^ Fourth, we defined trial eligibility based on BP measurements in routine practice. While this is a reasonable approximation of the population in whom the trial results will be applied, BP measurements in routine practice are more variable than those obtained in research settings.^35,36^ Fifth, the estimates produced by transportability analyses cannot be independently validated. Sixth, SPRINT was conducted before the widespread use of sodium glucose cotransporter 2 inhibitors, glucagon-like peptide-1 receptor agonists, and nonsteroidal mineralocorticoid receptor antagonists. Whether intensive BP treatment would result in similar benefits when achieved with pharmacotherapy regimens that differ from those used in SPRINT is unknown.

## Conclusions

In this comparative effectiveness study, while there were differences between participants enrolled in SPRINT and trial-eligible adults with CKD from the VHA and KPSC, we found that the trial results were transportable to the clinical populations and corresponded with similar or more favorable benefits on the absolute scale. Additionally, while intensive BP treatment was associated with similar benefits in adults with advanced CKD, this was incurred at the cost of an increased risk for certain adverse events, underscoring the importance of accounting for patient preferences in hypertension treatment decisions. This example highlights the potential for transportability methods to provide insights that can bridge evidence gaps and inform the application of novel therapies to patients with CKD who are treated in everyday practice.

## Notes

Supplement 1. **eMethods.** Description of Outcome-Based Model **eFigure 1.** Diagram of the Analytical Framework of the Outcome-Based Random Survival Forest Model **eFigure 2.** CONSORT Diagram of the Veteran Affairs Target Population **eFigure 3.** CONSORT Diagram of the Kaiser Permanente of Southern California Target Population **eTable 1.** *ICD* Diagnostic and CPT Procedure Codes Used to Define of Cardiovascular Event in the Target Population **eTable 2.** Risk Ratios at 4 Years of the Blood Pressure Treatment Intensive vs Standard in the SPRINT Trial and Transportability Estimate for the Target Populations, SPRINT-Eligible Adults With CKD in VA and KPSC for the Outcomes From the SPRINT Trials **eTable 3.** Risk Difference and Number Needed to Treat/Harm of the Blood Pressure Treatment Intensive vs Standard for SPRINT Trial and Transportability Estimate for the Target Populations, SPRINT-Eligible Adults With CKD in VA and KPSC for the Outcomes From the SPRINT Trial **eFigure 4.** Risk Ratio for the Primary and Secondary Outcomes and Adverse Events at 4 Years With Intensive vs Standard Blood Pressure Treatment in the SPRINT Population and Subsets of the Target Population, SPRINT Eligible Adults With an eGFR <45 mL/min/1.73 m^2^, and SPRINT Eligible Adults With an eGFR <30 mL/min/1.73m^2^, Stratified by Health Systems **eTable 4.** Risk Ratios at 4 Years of the Intensive vs Standard Blood Pressure Treatment in the SPRINT Trial and Transportability Estimate for the Subtarget Populations, SPRINT-Eligible Adults With CKD in VA and KPSC With eGFR <45 mL/min/1.73 m^2^ and eGFR <30 mL/min/1.73 m^2^ for the Outcomes From the SPRINT Trials
eReferences.

Supplement 2.
Data Sharing Statement
