# Supplementary material for: SPRINT Treatment Among Adults With Chronic Kidney Disease From 2 Large Health Care Systems
Source: JAMA Netw Open. 2025 Jan 7;8(1):e2453458. doi: 10.1001/jamanetworkopen.2024.53458 (PMC11707627; doi:10.1001/jamanetworkopen.2024.53458)
Supplement: Supplement 1. — eMethods. Description of Outcome-Based Model eFigure 1. Diagram of the Analytical Framework of the Outcome-Based Random Survival Forest Model eFigure 2. CONSORT Diagram of the Veteran Affairs Target Population eFigure 3. CONSORT Diagram of the Kaiser Permanente of Southern California Target Population eTable 1. ICD Diagnostic and CPT Procedure Codes Used to Define of Cardiovascular Event in the Target Population eTable 2. Risk Ratios at 4 Years of the Blood Pressure Treatment Intensive vs Standard in the SPRINT Trial and Transportability Estimate for the Target Populations, SPRINT-Eligible Adults With CKD in VA and KPSC for the Outcomes From the SPRINT Trials eTable 3. Risk Difference and Number Needed to Treat/Harm of the Blood Pressure Treatment Intensive vs Standard for SPRINT Trial and Transportability Estimate for the Target Populations, SPRINT-Eligible Adults With CKD in VA and KPSC for the Outcomes From the SPRINT Trial eFigure 4. Risk Ratio for the Primary and Secondary Outcomes and Adverse Events at 4 Years With Intensive vs Standard Blood Pressure Treatment in the SPRINT Population and Subsets of the Target Population, SPRINT Eligible Adults With an eGFR <45 mL/min/1.73 m2, and SPRINT Eligible Adults With an eGFR <30 mL/min/1.73m2, Stratified by Health Systems eTable 4. Risk Ratios at 4 Years of the Intensive vs Standard Blood Pressure Treatment in the SPRINT Trial and Transportability Estimate for the Subtarget Populations, SPRINT-Eligible Adults With CKD in VA and KPSC With eGFR <45 mL/min/1.73 m2 and eGFR <30 mL/min/1.73 m2 for the Outcomes From the SPRINT Trials eReferences. [file jamanetwopen-e2453458-s001.pdf]

Supplemental Online Content

Kurella Tamura M, Huang M, An J, et al. SPRINT treatment among adults with chronic kidney disease from 2 large health care systems. *JAMA Netw Open*. 2025;8(1):e2453458. doi:10.1001/jamanetworkopen.2024.53458

eMethods. Description of Outcome-Based Model
eFigure 1. Diagram of the Analytical Framework of the Outcome-Based Random Survival Forest Model
eFigure 2. CONSORT Diagram of the Veteran Affairs Target Population
eFigure 3. CONSORT Diagram of the Kaiser Permanente of Southern California Target Population
eTable 1. ICD Diagnostic and CPT Procedure Codes Used to Define of Cardiovascular Event in the Target Population
eTable 2. Risk Ratios at 4 Years of the Blood Pressure Treatment Intensive vs Standard in the SPRINT Trial and Transportability Estimate for the Target Populations, SPRINT-Eligible Adults With CKD in VA and KPSC for the Outcomes From the SPRINT Trials
eTable 3. Risk Difference and Number Needed to Treat/Harm of the Blood Pressure Treatment Intensive vs Standard for SPRINT Trial and Transportability Estimate for the Target Populations, SPRINT-Eligible Adults With CKD in VA and KPSC for the Outcomes From the SPRINT Trial
eFigure 4. Risk Ratio for the Primary and Secondary Outcomes and Adverse Events at 4 Years With Intensive vs Standard Blood Pressure Treatment in the SPRINT Population and Subsets of the Target Population, SPRINT Eligible Adults With an eGFR <45 mL/min/1.73 m2, and SPRINT Eligible Adults With an eGFR <30 mL/min/1.73m2, Stratified by Health Systems
eTable 4. Risk Ratios at 4 Years of the Intensive vs Standard Blood Pressure Treatment in the SPRINT Trial and Transportability Estimate for the Subtarget Populations, SPRINT-Eligible Adults With CKD in VA and KPSC With eGFR <45 mL/min/1.73 m2 and eGFR <30 mL/min/1.73 m2 for the Outcomes From the SPRINT Trials
eReferences.

This supplemental material has been provided by the authors to give readers additional information about their work.

© 2024 Kurella Tamura M et al. *JAMA Network Open*.

eMethods. Description of outcome-based model

We first re-estimated the average treatment effect in the SPRINT trial, calculating the counterfactual risk ratio (RR) at 4 years for each outcome. For this purpose, we used a flexible parametric survival model[1,2], which is fitted on the log cumulative excess hazard scale, using restricted cubic splines to estimate the baseline cumulative excess hazard. We then predicted the survival probability of the potential outcome $Y^*$of the trial individual i (where $i \in \{1{:}\,n\}$, and n is the total number of the trial participants) assuming they are all treated or not treated, denoted as $S_j(Y^*|a = 1)$, $S_j(Y^*|a = 0)$ respectively, where a is the indicator for treatment arm. Then we calculate the sample average treatment effect as a risk ratio (RR_{SATE}) based on Eq. 1.

$$RR_{SATE} = \frac{1-(\frac{\sum_{i=1}^{n} s_i(Y^*|a=1)}{n})}{1-(\frac{\sum_{i=1}^{n} s_i(Y^*|a=0)}{n})} \qquad (1)$$

Due to the small sample size ratio of the SPRINT trial versus the target populations, we used an outcome-based approach which is particularly effective when effect modifiers are strong predictors of the outcome and when the outcome is common but selection into the study is rare[3]. The general analytical framework is shown in **eFigure 1**. The outcome Y was modeled separately for each treatment arm using the trial data. Predictors for the two models were the observed potential effect modifiers: baseline age (years), sex (female vs. male), race/ethnicity (non-Hispanic white, non-Hispanic black, Hispanic, and other), cardiovascular disease history (present of one of the seven ICD and/or CPT-defined cardiovascular conditions, including myocardial Infarction, acute coronary syndrome, coronary artery bypass surgery, percutaneous coronary intervention, carotid endarterectomy, peripheral vascular disease with revascularization and AAA repair, e**Table 1)** in the past 24 months (yes vs. no), systolic BP (mmHg), diastolic BP (mmHg), urine albumin/creatinine ratio (UACR, mg/g), eGFR (mL/min/1.72m^2), Framingham Risk Score, body mass index (BMI, kg/m^2), number of BP medications, high-density lipoprotein cholesterol (HDL, mg/dL), low-density lipoprotein cholesterol (LDL, mg/dL), ever smoker (yes vs. no), and statin use (yes vs. no). The fitted models were then used to predict the potential outcomes Y* in the target population, from which target average treatment effect (TATE) were estimated by averaging the individual causal effects obtained from the difference/ratio of predicted potential outcomes[4]. The analytical outcome-based model applied was a random survival forest model built based on the work by Lu et al[5] on causal RF[6]. Like the calculation of RR_{SATE}, we calculated the counterfactual RR at 4 years for the TATE based on Eq.2. We performed 1000 bootstraps for RR_{SATE} and 150 bootstraps for RR_{TATE} to calculate 95% confidence intervals (CIs).

$$RR_{TATE} = \frac{1-(\frac{\sum_{j=1}^{N} s_j(Y^*|a=1)}{N})}{1-(\frac{\sum_{j=1}^{N} s_j(Y^*|a=0)}{N})} \qquad (2),$$

where $S_j(Y^*|a = 1)$ is the predicted survival probability of the potential outcome Y^* for the jth individual in the target population ($j \in \{1{:}\,N\}$, N is the size of the target population) assuming they are treated; and $S_j(Y^*|a = 0)$ is the predicted survival probability of the potential outcome Y^* for the jth individual in the target population assuming they are not treated.

© 2024 Kurella Tamura M et al. *JAMA Network Open*.

Discussion and evaluation of transportability assumptions

This analysis comes with a large set of assumptions: applied to the trial itself that 1) guarantee internal validity and 2) necessary for external validity, and 3) required for transportability of the trial results into a target population of interest.

In the following table we describe each assumption and discuss its applicability in the current study.

Transportability Assumption	Discussion
**Required for internal validity of the original study (SPRINT trial)**	
**Conditional treatment exchangeability**: no unmeasured confounding of the treatment-outcome relationship in the trial.	Assumed because of randomization.
**Positivity of treatment assignment**: that each individual in the trial has a positive probability of receiving either treatment.	Assumed because of randomization.
**Stable unit treatment value assumption (SUTVA) that requires non-interference between subjects and treatment**: that the potential outcome of a subject is unaffected by the treatment assignment of other subjects in both the trial and target populations.	No interference in the context of a clinical trial implies that the trial's conditions, treatments, and outcomes are not affected by outside influences. The SPRINT trial was conducted under a controlled environment with an inclusion and exclusion criteria for participant selection that ensured that the treatment effect can be attributed to the blood pressure control regimens being tested. Monitoring processes were in place that ensure compliance with the study protocol, with regular check-ins with the participants to maintain adherence to the treatment regimens and prevent deviations that could affect the outcomes.
**Necessary for external validity**	
**Conditional exchangeability for study selection**: that the outcomes among individuals with the same treatment and covariate values in the trial and target populations are the same.	Direct comparison of outcomes in the trial and the target population is difficult because, in the target population, defined using claims data, outcomes are not adjudicated, and reporting does not follow a strict protocol. This increases variability. However, our main target population is one that meets the inclusion and exclusion criteria for the SPRINT trial. As such we would expect that the outcome distribution is similar between the trial sample and target population.
**Positivity of selection** requires a common support with respect to study selection: that	This assumption is met for the main target population given that it was selected to

© 2024 Kurella Tamura M et al. *JAMA Network Open*.

in every stratum of effect modifiers, there is a positive probability of being in the study sample. This assumption can be relaxed to a conditional positivity of selection where we assume that all members of the target population are represented by individuals in the trial.	meet the inclusion and exclusion criteria.
**SUTVA for study selection:** no interference between subjects selected and not selected into the trial.	Given the difference in time frame from when the trial was conducted and when the study population was defined there was no interference between the trial participants and those in the target population.
**Required for transportability**	
**Conditional exchangeability for study selection under a transportability scenario**: the range of values for each treatment effect modifier considered in the target population must be within the range of those in the trial population.	Met since our main target population is one that meets the inclusion and exclusion criteria for the SPRINT trial.

© 2024 Kurella Tamura M et al. *JAMA Network Open*.

eFigure 1. Diagram of the analytical framework of the outcome-based random survival forest model

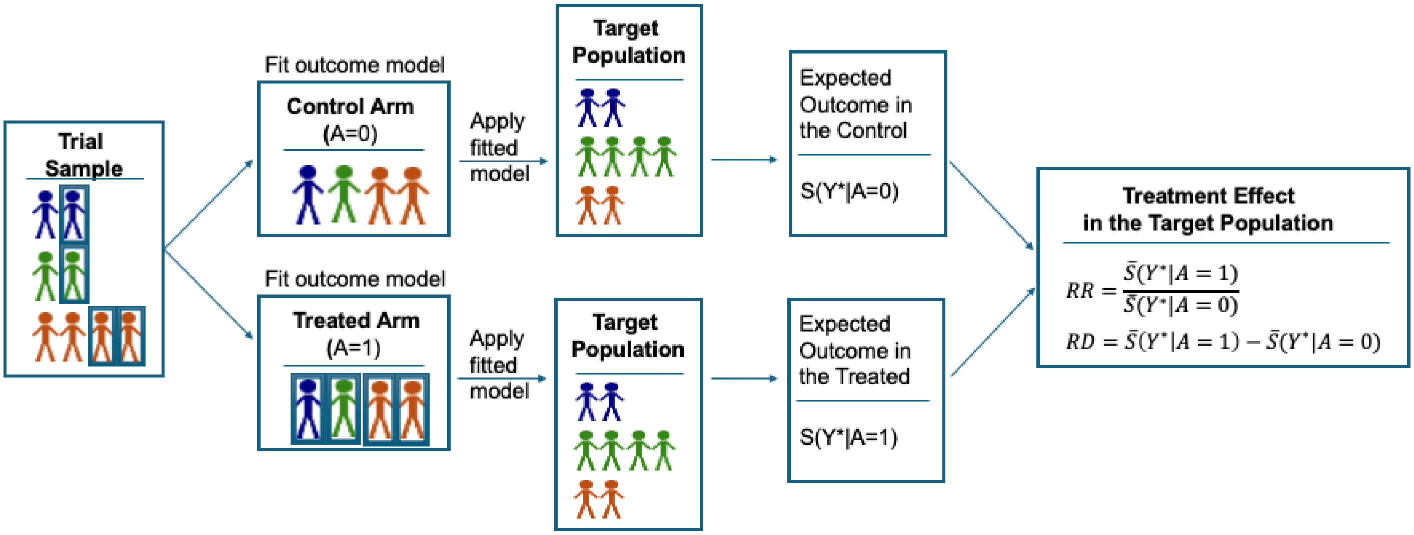

© 2024 Kurella Tamura M et al. *JAMA Network Open*.

eFigure 2. CONSORT diagram of the Veteran Affairs target population

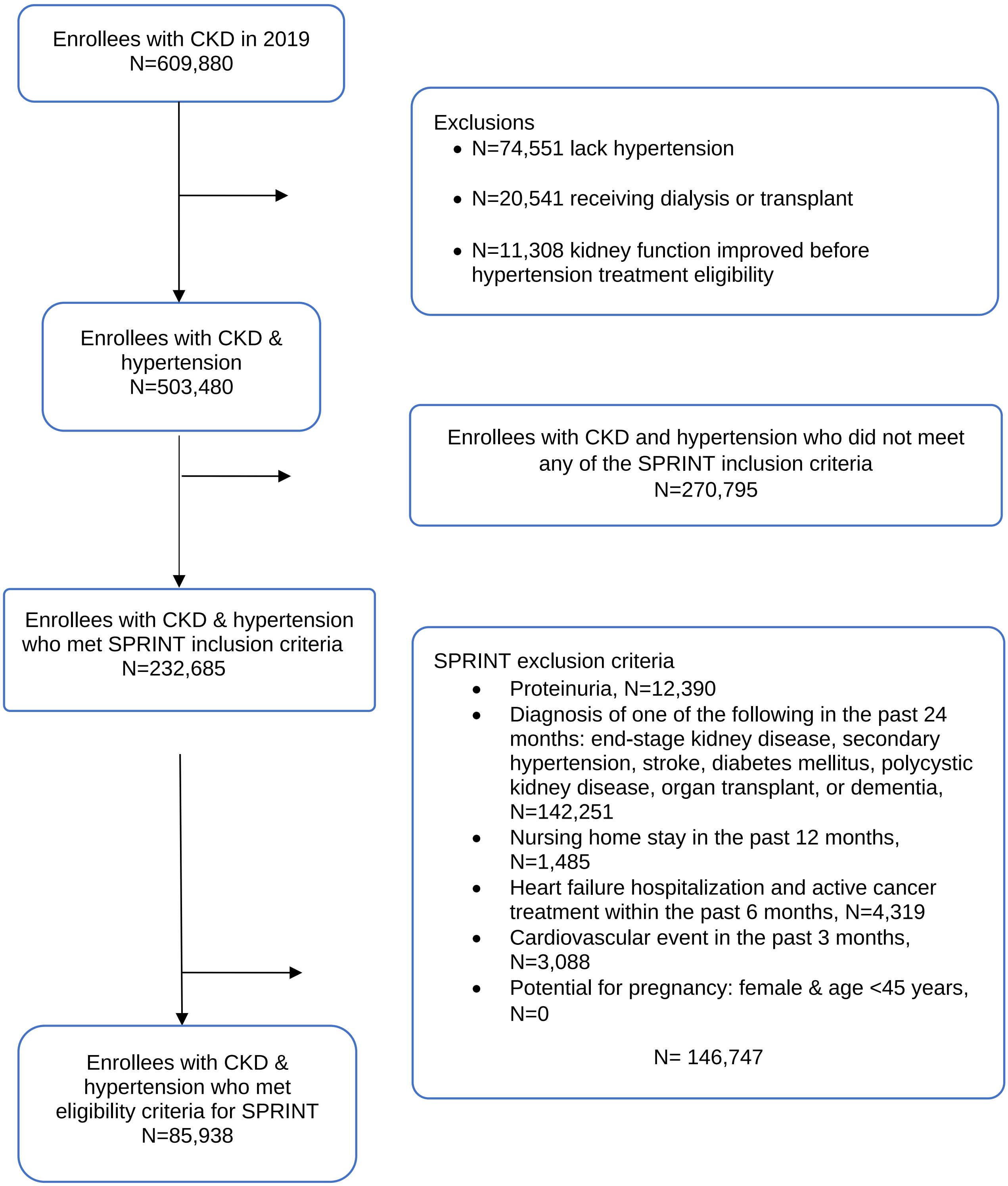

© 2024 Kurella Tamura M et al. *JAMA Network Open*.

eFigure 3. CONSORT diagram of the Kaiser Permanente of Southern California target population

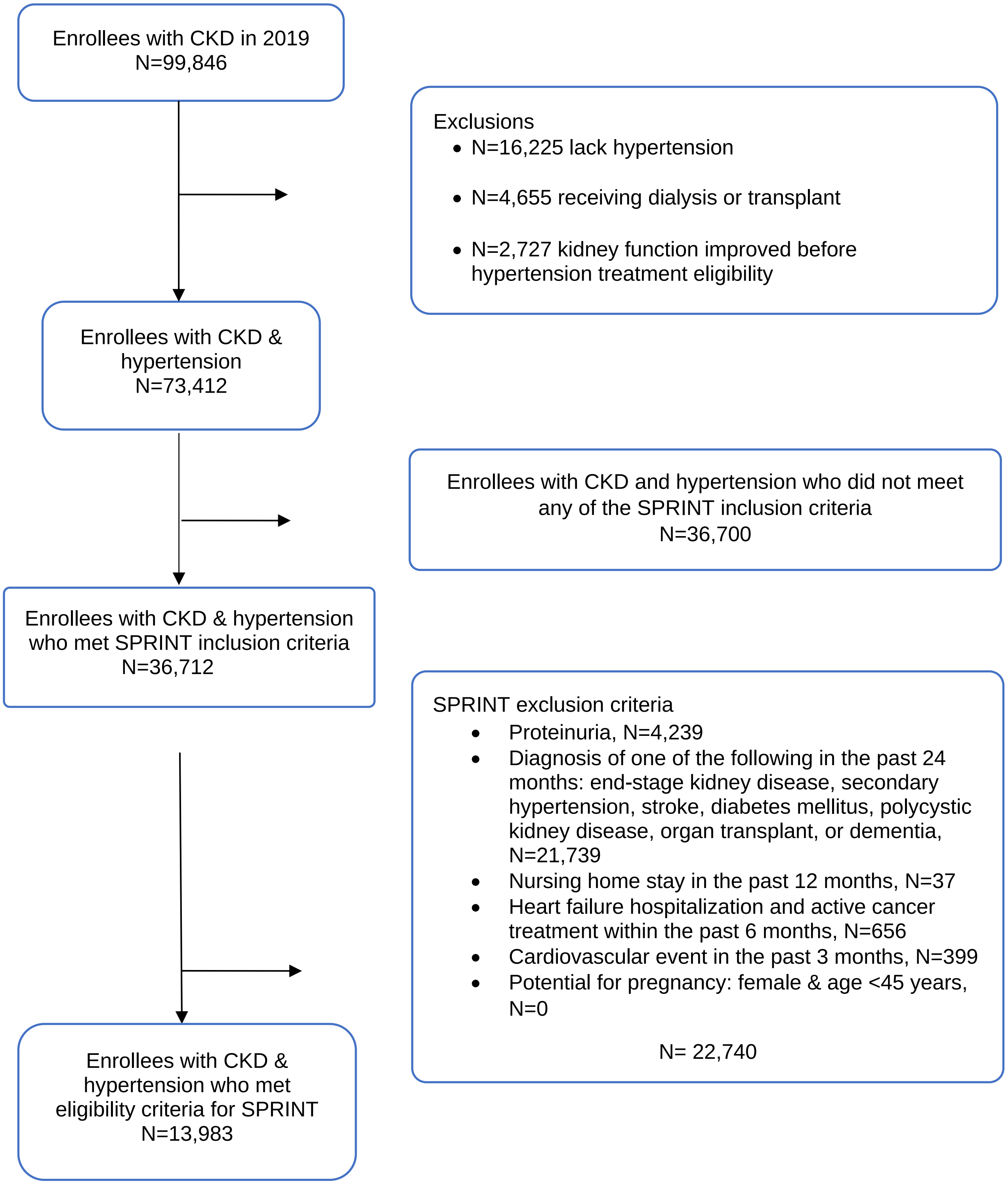

© 2024 Kurella Tamura M et al. *JAMA Network Open*.

eTable 1. ICD diagnostic and CPT procedure codes used to define of cardiovascular event in the target population.

Condition name	ICD-9	ICD-10	CPT
Myocardial Infarction	410, 410.01, 410.02, 410.1, 410.11, 410.12, 410.2, 410.21, 410.22, 410.3, 410.31, 410.32, 410.4, 410.41, 410.42, 410.5, 410.51, 410.52, 410.6, 410.61, 410.62, 410.7, 410.71, 410.72, 410.8, 410.81, 410.82, 410.9, 410.91, 410.92	I21.01, I21.02, I21.09, I21.11, I21.19, I21.21, I21.29, I21.3, I21.4, I21.9, I21.A1, I21.A9, I22.0, I22.1, I22.2, I22.8, I22.9	
acute coronary syndrome	411, 411.1, 411.8, 411.81, 411.89, 413, 413.9, 414, 414.01, 414.02, 414.03, 414.04, 414.05, 414.06, 414.07, 414.2, 414.3, 414.4, 414.8, 414.9	I24.0, I24.8, I24.9	
coronary artery bypass surgery	36.03, 36.1, 36.11, 36.12, 36.13, 36.14, 36.15, 36.16, 36.17, 36.19, 36.2, 36.31, 36.32, 36.33	02100xx, 02103xx, 02104xx, 02110xx, 02113xx, 02114xx, 02120xx, 02123xx, 02124xx, 02130xx, 02133xx, 02134xx, 021K0xx, 021L0xx, 02C00xx, 02C10xx, 02C20xx, 02C30xx, 3E070xx	33510, 33511, 33512, 33513, 33514, 33516, 33517, 33518, 33519, 33521, 33522, 33523, 33530, 33533, 33534, 33535, 33536
percutaneous coronary intervention	0.66	021K4xx, 021L4xx, 02710xx, 02720xx, 02723xx, 02724xx, 02730xx, 02733xx, 02734xx, 02C03xx, 02C04xx, 02C13xx, 02C14xx, 02C23xx, 02C24xx, 02C33xx, 02C34xx, 02QA4xx, 02QB4xx, 02QC4xx, 3E073xx	CPT: 92920, 92924, 92928, 92933, 92937, 92941, 92943, 92980, 92982, 92984, 92995, 92996;<br>HCPCS: C9600, C9602, C9604, C9606, C9607
carotid endarterectomy			35301

© 2024 Kurella Tamura M et al. *JAMA Network Open*.

peripheral vascular disease with revascularization			37184-89, 37236-37239, 35302-06,35331, 35381, 35452, 37220-37223, 37224-37227, 37228-37234, 37235, 35472, 35481-2, 35491, 0238T, 35521, 35533, 35537-41, 35546, 35548-49, 35551, 35556, 35558, 35563, 35565-66, 35571, 35582-83, 35585, 35587, 35621, 35623, 35637-38, 35641,35646-47, 35651, 35654, 35656, 35661, 35663, 35665-66, 35671, 35351, 35355, 35361, 35363, 35371-72, 35226, 35256, 35286,35700, 35721, 35741, 35876, 35879, 35881, 35883, 35884
AAA repair			34800, 34802-34805, 35091-35092

© 2024 Kurella Tamura M et al. *JAMA Network Open*.

eTable 2. Risk ratios at four years and 95% confidence intervals of the blood pressure treatment intensive vs. standard in the SPRINT trial and transportability estimate for the target populations, SPRINT eligible adults with CKD in VA and KPSC for the outcomes from the SPRINT trials.

	Intensive Treatment n=4678			Standard Treatment n=4683			SATE (stratified by clinic site)	Target estimates VA N=85938	Target estimates KPSC N=13983
**Outcome**	no. of events	person time (person-yr)	incidence rate (percent / yr)	no. of events	person time (person-yr)	incidence rate (percent / yr)	Intensive vs. Standard RR [95%CI]		
Primary outcome	264	14307	1.85	354	14162	2.5	0.75 [0.63-0.86]	0.76 [0.66, 1.12]	0.8 [0.64-1.02]
All-cause Death	163	14686	1.11	215	14632	1.47	0.76 [0.63-0.93]	0.84 [0.63, 1.11]	0.83 [0.63-1.09]
Death from cardiovascular causes	41	14639	0.28	71	14585	0.49	0.58 [0.39-0.82]	0.78 [0.39, 1.37]	0.65 [0.4-1.16]
Primary outcome / all-cause death	370	14305	2.59	474	14160	3.35	0.78 [0.69-0.89]	0.81 [0.71, 1.08]	0.86 [0.7-1.05]
Primary outcome / cardiovascular procedure	306	14224	2.15	454	14034	2.9	0.75 [0.66-0.85]	0.78 [0.65, 1.15]	0.78 [0.67-1.03]
Primary outcome without non-fatal heart failure	222	14364	1.55	293	14247	2.06	0.76 [0.64-0.90]	0.74 [0.6, 1.04]	0.72 [0.57-1.01]
Myocardial infarction	101	14486	0.7	139	14388	0.97	0.72 [0.56-0.94]	0.62 [0.44, 1.09]	0.76 [0.48-1.17]
Acute coronary syndrome	42	14555	0.29	41	14497	0.28	1.02 [0.66-1.61]	2.42 [1.2, 5.22]	1.17 [0.62-2.27]

© 2024 Kurella Tamura M et al. *JAMA Network Open*.

Stroke	68	14553	0.47	76	14474	0.53	0.89 [0.63-1.24]	0.59 [0.4, 1.13]	0.74 [0.48-1.17]
Heart failure	66	14557	0.45	101	14447	0.7	0.65 [0.48-0.88]	0.73 [0.48, 1.41]	0.92 [0.57-1.34]
Dementia	149	20613	0.72	176	20422	0.86	0.84 [0.68-1.04]	1.13 [0.84, 2.12]	1.06 [0.78-1.5]
Mild cognitive impairment	287	19731	1.45	353	19321	1.83	0.81 [0.71-1.08]	1.06 [0.89, 1.43]	1.04 [0.82-1.27]
Composite kidney outcome[a]	17	3838	0.44	16	3694	0.43	1.04 [0.53-3.08]	0.33 [0.17, 1.36]	1.09 [0.4-3.11]
eGFR >50% reduction[a]	12	3829	0.31	12	3656	0.33	0.98 [0.47-6.76]	0.29 [0.11, 1.25]	1.03 [0.33-3.81]
Dialysis or transplant[a]	7	4171	0.17	10	4109	0.24	0.69 [0.21-2.01]	0.18 [0.07, 1.75]	0.62 [0.22-2.3]
Any serious adverse event	802	13340	6.01	640	13607	4.7	1.24 [1.14-1.37]	1.04 [0.88, 1.21]	1.11 [0.95-1.31]
Hypotension	143	14392	0.99	79	14435	0.55	1.80 [1.40-2.37]	2.04 [1, 3.04]	1.56 [0.95-2.84]
Syncope	148	14383	1.03	100	14427	0.69	1.47 [1.17-1.94]	1.7 [1.01, 2.46]	1.38 [0.96-2.1]
Bradycardia	93	14485	0.64	76	14457	0.53	1.22 [0.92-1.64]	1.2 [0.7, 2.03]	1.12 [0.69-1.8]
Electrolyte abnormality	170	14335	1.19	127	14379	0.88	1.33 [1.05-1.67]	1.36 [0.85, 2.09]	1.24 [0.83-1.74]
Injurious fall	334	14141	2.36	317	14111	2.25	1.05 [0.90-1.22]	0.82 [0.61, 1.04]	1.17 [0.81-1.53]
Acute kidney injury	200	14343	1.39	120	14419	0.83	1.65 [1.29-2.06]	1.73 [1.25, 2.92]	1.66 [1.23-2.34]

[a] The sample average treatment effect in the trial for the kidney outcomes (eGFR >50% reduction and Dialysis or transplant) were estimated among the SPRINT CKD subgroup.

Abbreviations: SPRINT – Systolic Pressure Intervention Trial, CKD – chronic kidney disease; VA – Veterans Health Administration, KPSC – Kaiser Permanente of Southern California; SATE – sample average treatment effect; RR– risk ratio; 95%CI – 95% confidence interval; eGFR – estimated glomerular filtration rate.

© 2024 Kurella Tamura M et al. *JAMA Network Open*.

eTable 3. Risk ratios at four years and 95% confidence intervals of the blood pressure treatment intensive vs. standard in the SPRINT trial and transportability estimate for the sub-target populations, SPRINT eligible adults with CKD in VA and KPSC whose eGFR <45mL/min/1.73m^2 and eGFR<30mL/min/1.73m^2 for the outcomes from the SPRINT trials.

	SATE RR [95%CI]	VA RR [95%CI]		KPSC RR [95%CI]	
		eGFR<45 N=24,478	eGFR<30 N=3,558	eGFR<45 N=4,232	eGFR<30 N=599
Outcome					
Primary outcome	0.75 [0.63-0.86]	0.84 [0.65, 1.1]	0.86 [0.68, 1.16]	0.88 [0.66, 1.14]	0.88 [0.66, 1.19]
All-cause Death	0.76 [0.63-0.93]	0.84 [0.63, 1.09]	0.81 [0.61, 1.12]	0.82 [0.62, 1.12]	0.77 [0.57, 1.14]
Death from cardiovascular causes	0.58 [0.39-0.82]	0.79 [0.42, 1.38]	0.71 [0.4, 1.32]	0.69 [0.40, 1.20]	0.63 [0.38, 1.11]
Primary outcome/ all-cause death	0.78 [0.69-0.89]	0.87 [0.71, 1.07]	0.88 [0.72, 1.1]	0.91 [0.72, 1.13]	0.89 [0.70, 1.15]
Primary outcome/cardiovascular procedure	0.75 [0.66-0.85]	0.87 [0.69, 1.11]	0.91 [0.71, 1.17]	0.86 [0.71, 1.15]	0.91 [0.73, 1.22]
Primary outcome without non-fatal heart failure	0.76 [0.64-0.90]	0.72 [0.58, 1.02]	0.74 [0.58, 1.05]	0.70 [0.54, 0.97]	0.65 [0.51, 0.95]
Myocardial infarction	0.72 [0.56-0.94]	0.67 [0.43, 1.09]	0.7 [0.43, 1.16]	0.77 [0.45, 1.20]	0.76 [0.44, 1.25]
Acute coronary syndrome	1.02 [0.66-1.61]	2.31 [1.01, 4.9]	2.83 [1.21, 5.74]	1.23 [0.61, 2.33]	1.67 [0.73, 3.27]
Stroke	0.89 [0.63-1.24]	0.6 [0.38, 1.06]	0.7 [0.39, 1.16]	0.66 [0.43, 1.13]	0.63 [0.39, 1.08]
Heart failure	0.65 [0.48-0.88]	0.93 [0.57, 1.42]	1 [0.64, 1.53]	1.14 [0.72, 1.68]	1.28 [0.78, 1.94]
Dementia	0.84 [0.68-1.04]	1.3 [0.9, 1.92]	1.54 [1.03, 2.24]	1.18 [0.84, 1.67]	1.49 [0.98, 2.11]
Mild cognitive impairment	0.81 [0.71-1.08]	1.11 [0.87, 1.39]	1.17 [0.91, 1.48]	1.10 [0.86, 1.35]	1.18 [0.89, 1.47]
Composite kidney outcome	1.04 [0.53-3.08]	0.35 [0.16, 1.26]	0.46 [0.21, 1.31]	1.02 [0.43, 2.67]	0.90 [0.44, 1.94]
eGFR >50% reduction	0.98 [0.47-6.76]	0.26 [0.09, 1.15]	0.27 [0.1, 1.07]	0.98 [0.30, 3.24]	0.75 [0.29, 2.08]

© 2024 Kurella Tamura M et al. *JAMA Network Open*.

Dialysis or transplant	0.69 [0.21-2.01]	0.22 [0.06, 1.56]	0.43 [0.15, 1.89]	0.64 [0.23, 2.32]	0.86 [0.27, 2.70]
Any serious adverse event	1.24 [1.14-1.37]	0.97 [0.84, 1.17]	1.01 [0.87, 1.21]	1.01 [0.85, 1.26]	1.07 [0.89, 1.30]
Hypotension	1.80 [1.40-2.37]	1.7 [1.04, 2.95]	1.57 [0.94, 2.89]	1.21 [0.71, 2.75]	1.20 [0.69, 2.81]
Syncope	1.47 [1.17-1.94]	1.71 [1.08, 2.55]	1.62 [1, 2.47]	1.35 [0.93, 2.13]	1.36 [0.85, 2.16]
Bradycardia	1.22 [0.92-1.64]	1.08 [0.61, 1.96]	1.13 [0.66, 2.02]	1.10 [0.66, 1.78]	1.18 [0.68, 1.96]
Electrolyte abnormality	1.33 [1.05-1.67]	1.44 [0.86, 2.16]	1.56 [0.89, 2.27]	1.27 [0.81, 1.79]	1.34 [0.84, 1.90]
Injurious fall	1.05 [0.90-1.22]	0.75 [0.58, 0.98]	0.81 [0.61, 1.05]	1.15 [0.73, 1.49]	1.21 [0.74, 1.57]
Acute kidney injury	1.65 [1.29-2.06]	2.22 [1.36, 2.91]	2.34 [1.45, 3.1]	2.01 [1.39, 2.87]	2.10 [1.43, 3.03]

Abbreviations: SPRINT – Systolic Pressure Intervention Trial, CKD – chronic kidney disease; VA – Veterans Health Administration, KPSC – Kaiser Permanente of Southern California; SATE – sample average treatment effect; 95%CI – 95% confidence interval; eGFR – estimated glomerular filtration rate.

© 2024 Kurella Tamura M et al. *JAMA Network Open*.

eTable 4. Risk difference and 95% confidence intervals and number needed to treat/harm of the blood pressure treatment intensive vs. standard for SPRINT trial and transportability estimate for the target populations, SPRINT eligible adults with CKD in VA and KPSC for the outcomes from the SPRINT trial.

	SATE		Target estimates VA		Target estimates KPSC	
Outcome	NNT(+)/ NNH(-)[a]	RD,% [95%CI]	NNT(+)/ NNH(-)[a]	RD,% [95%CI]	NNT(+)/ NNH(-)[a]	RD,% [95%CI]
Primary outcome	41	-2.4 [-3.8, -1.2]	20	-5.1 [-9.8, 3.2]	33	-3 [-6.3, 0.3]
All-cause Death	64	-1.6 [-2.6, -0.4]	36	-2.8 [-11.7, 2.5]	44	-2.3 [-5.7, 1]
Death from cardiovascular causes	110	-0.9 [-1.5, -0.3]	59	-1.7 [-9, 3]	69	-1.4 [-3.6, 0.6]
Primary outcome/ all-cause death	37	-2.7 [-4, -1.3]	20	-5.1 [-10.4, 2.7]	33	-3.1 [-6.6, 0.9]
Primary outcome/cardiovascular procedure	36	-2.8 [-4, -1.5]	19	-5.3 [-9.8, 4.1]	34	-2.9 [-6.2, 0.4]
Primary outcome without non-fatal heart failure	50	-2 [-3.2, -0.8]	21	-4.7 [-10.8, 0.8]	30	-3.3 [-6, 0.1]
Myocardial infarction	91	-1.1 [-2, -0.2]	25	-4 [-9.2, 1.2]	65	-1.5 [-3.7, 0.9]
Acute coronary syndrome	-4424	0 [-0.5, 0.5]	-59	1.7 [0.3, 4.7]	-563	0.2 [-0.6, 0.9]
Stroke	423	-0.2 [-0.9, 0.4]	36	-2.8 [-7, 1.2]	80	-1.2 [-3, 0.6]
Heart failure	94	-1.1 [-1.8, -0.3]	37	-2.7 [-6.6, 5.3]	235	-0.4 [-3.6, 1.9]
Dementia	208	-0.5 [-1.1, 0.1]	-111	0.9 [-1.3, 9.5]	-212	0.5 [-2.1, 3.4]
Mild cognitive impairment	64	-1.6 [-2.5, 0.1]	-100	1 [-2.3, 8.6]	-136	0.7 [-3.8, 4.8]
Composite kidney outcome	-1331	0.1 [-1.1, 1.3]	32	-3.1 [-9.9, 1.3]	-557	0.2 [-2.4, 2.5]
eGFR >50% reduction	3179	0 [-1.2, 1.1]	31	-3.2 [-12.3, 0.5]	5399	0 [-2.5, 2.2]
Dialysis or transplant	371	-0.3 [-1.0, 0.5]	63	-1.6 [-7.2, 1.1]	430	-0.2 [-1, 0.3]

© 2024 Kurella Tamura M et al. *JAMA Network Open*.

Any serious adverse event	-23	4.3 [2.5, 6.1]	-77	1.3 [-5.5, 7.7]	-33	3.1 [-1.5, 8.3]
Hypotension	-60	1.7 [1, 2.4]	-23	4.3 [0, 7.7]	-51	2 [-0.2, 4.1]
Syncope	-75	1.3 [0.5, 2.3]	-29	3.4 [0.1, 7.4]	-44	2.2 [-0.3, 4.6]
Bradycardia	-218	0.5 [-0.2, 1.2]	-83	1.2 [-3.2, 6]	-170	0.6 [-1.7, 3.3]
Electrolyte abnormality	-86	1.2 [0.2, 2.1]	-50	2 [-1.2, 7.9]	-97	1.0 [-1.2, 3.3]
Injurious fall	-229	0.4 [-1, 1.9]	29	-3.5 [-11.6, 0.6]	-55	1.8 [-3.2, 6.2]
Acute kidney injury	-45	2.2 [1.1, 3.2]	-13	7.5 [3.3, 26.1]	-19	5.3 [2.2, 8.7]

[a] NNT or NNH equals to -100 divided by the risk difference (%).

Abbreviations: SPRINT – Systolic Pressure Intervention Trial, CKD – chronic kidney disease; VA – Veterans Health Administration, KPSC – Kaiser Permanente of Southern California; SATE – sample average treatment effect; NNT – number needed to treat; NNH – number needed to harm; RD – risk difference; 95%CI – 95% confidence interval; eGFR – estimated glomerular filtration rate.

© 2024 Kurella Tamura M et al. *JAMA Network Open*.

References

1. Nelson CP, Lambert PC, Squire IB, Jones DR. Flexible parametric models for relative suvival, with application in coronary heart disease. In: *Statistics in Medicine*. Vol 26. ; 2007. doi:10.1002/sim.3064
2. Lambert PC, Royston P. Further development of flexible parametric models for survival analysis. *Stata Journal*. 2009;9(2). doi:10.1177/1536867x0900900206
3. Degtiar I, Rose S. A Review of Generalizability and Transportability. *Annu Rev Stat Appl*. 2023;10. doi:10.1146/annurev-statistics-042522-103837
4. Hernán MA, Robins JM. Estimating causal effects from epidemiological data. *J Epidemiol Community Health (1978)*. 2006;60(7). doi:10.1136/jech.2004.029496
5. Lu M, Sadiq S, Feaster DJ, Ishwaran H. Estimating Individual Treatment Effect in Observational Data Using Random Forest Methods. *Journal of Computational and Graphical Statistics*. 2018;27(1). doi:10.1080/10618600.2017.1356325
6. Goldstein BA, Phelan M, Pagidipati NJ, Holman RR, Pencina MJ, Stuart EA. An outcome model approach to transporting a randomized controlled trial results to a target population. *Journal of the American Medical Informatics Association*. 2019;26(5). doi:10.1093/jamia/ocy188
7. Robins JM, Hernán MÁ, Brumback B. Marginal structural models and causal inference in epidemiology. *Epidemiology*. 2000;11(5). doi:10.1097/00001648-200009000-00011
8. Cole SR, Frangakis CE. The consistency statement in causal inference: A definition or an assumption? *Epidemiology*. 2009;20(1). doi:10.1097/EDE.0b013e31818ef366

© 2024 Kurella Tamura M et al. *JAMA Network Open*.
