# Supplementary material for: SPRINT Treatment Among Adults With Chronic Kidney Disease From 2 Large Health Care Systems
Source: JAMA Netw Open. 2025 Jan 7;8(1):e2453458. doi: 10.1001/jamanetworkopen.2024.53458 (PMC11707627; doi:10.1001/jamanetworkopen.2024.53458)
Supplement: Supplement 2. — Data Sharing Statement [file jamanetwopen-e2453458-s002.pdf]

Data Sharing Statement

Kurella Tamura. SPRINT Treatment Among Adults With Chronic Kidney Disease From 2 Large Health Care Systems. *JAMA Netw Open*. Published January 07, 2025. doi:10.1001/jamanetworkopen.2024.53458

Data
Data available: No
